# Lipoprotein(a) as a Risk Factor for Recurrent Acute Myocardial Infarction and Mortality: Insights from Routine Clinical Practice

**DOI:** 10.3390/diagnostics14232757

**Published:** 2024-12-07

**Authors:** David Šuran, Vojko Kanič, Peter Kokol, Tadej Završnik, Florjan Verhnjak, Bojan Žlahtič, Andreja Sinkovič, Franjo Husam Naji

**Affiliations:** 1 Clinical Department of Cardiology and Angiology, University Medical Centre Maribor, Ljubljanska ulica 5, 2000 Maribor, Slovenia; vojko.kanic@guest.arnes.si (V.K.); tadej.zavrsnik@student.um.si (T.Z.); florjan.verhnjak@gmail.com (F.V.); franjo.naji@ukc-mb.si (F.H.N.); 2 Faculty of Medicine, University of Maribor, Taborska ulica 8, 2000 Maribor, Slovenia; peter.kokol@um.si (P.K.); andreja.sinkovic@guest.arnes.si (A.S.); 3 Faculty of Electrical Engineering and Computer Science, University of Maribor, Koroška cesta 46, 2000 Maribor, Slovenia; bojan.zlahtic@um.si; 4 Department of Medical Intensive Care, University Medical Centre Maribor, Ljubljanska ulica 5, 2000 Maribor, Slovenia

**Keywords:** lipoprotein(a), risk factor, acute myocardial infarction, cardiovascular mortality, all-cause mortality, women

## Abstract

Background: Lipoprotein(a) [Lp(a)] is a well-established risk factor for incident atherosclerotic cardiovascular (CV) disease. However, evidence regarding its association with recurrent events is limited. To address this gap, we conducted a retrospective analysis of routine clinical data, focusing on patients hospitalized for acute myocardial infarction (AMI) between 2000 and 2022 with available admission Lp(a) results. Methods: Patients were stratified into three groups based on their Lp(a) level (≤50 mg/dL, 51–90 mg/dL, and >90 mg/dL). A multivariable-adjusted Cox regression analysis was performed to assess the associations of Lp(a) with recurrent AMI, CV mortality, and all-cause mortality. Results: A total of 2248 patients (31.5% women), with a mean age of 64.7 ± 12.2 years, were retrospectively followed until 31 December 2022, or death. The multivariable-adjusted hazard ratios (HRs) for recurrent AMI were 1.01 (*p* = 0.921) for levels 51–90 mg/dL and 1.51 (*p* = 0.013) for levels > 90 mg/dL, compared with levels ≤ 50 mg/dL. The corresponding HRs for CV mortality were 1.13 (*p* = 0.300) and 1.14 (*p* = 0.348), and those for all-cause mortality were 1.09 (*p* = 0.310) and 1.20 (*p* = 0.090), respectively. Stratification by sex and age revealed a significant association of Lp(a) with recurrent AMI only in women aged > 65 years, with adjusted HRs of 2.34 (*p* = 0.013) for levels 51–90 mg/dL and 3.94 (*p* < 0.001) for levels > 90 mg/dL, compared with levels ≤ 50 mg/dL. Conclusions: In the presented study, Lp(a) was associated with a significantly higher risk of recurrent AMI only in women aged > 65 years with Lp(a) levels > 50 mg/dL. We found no significant associations between Lp(a) and CV or all-cause mortality.

## 1. Introduction

Lipoprotein(a) [Lp(a)] is a lipoprotein particle structurally similar to low-density lipoprotein (LDL) with an additional apolipoprotein(a) [apo(a)] covalently attached to apolipoprotein B. Its serum concentration is primarily determined by genetics and is not significantly affected by dietary or lifestyle changes [1]. Lp(a) is prone to oxidative modifications and possesses pro-inflammatory and pro-atherogenic properties, and due to its similarity to plasminogen, it may also exert potential pro-thrombotic effects [2]. It is a well-recognized causal risk factor for atherosclerotic cardiovascular disease (ASCVD) and calcific aortic valve stenosis [3,4,5,6,7,8,9,10].

The European Society of Cardiology (ESC)/European Atherosclerosis Society (EAS) guidelines recommend screening for Lp(a) at least once during adulthood [11]. However, Lp(a) levels tend to increase with age. While the rise is generally steady in men, most studies have reported a modest additional increase in women around the time of menopause [2,11,12].

Numerous studies have confirmed the association of Lp(a) with incident cardiovascular (CV) events, including those in coronary, cerebrovascular, and peripheral arterial territories [5,6,7,13,14,15,16,17,18]. On the other hand, its role in patients with established ASCVD remains controversial, with varying findings in previous reports [7,19,20,21,22]. Extensive analysis of Lp(a) data from the UK Biobank suggested that the impact of Lp(a) on CV events is attenuated in secondary prevention compared with primary prevention settings [7]. To further investigate Lp(a) as a risk factor for recurrent acute myocardial infarction (AMI) and mortality, we conducted a retrospective analysis of data from our clinical routine.

## 2. Materials and Methods

### 2.1. Patients

Using the Tenth Revision of the International Classification of Diseases (ICD-10) diagnosis codes, we identified all patients admitted for AMI to the Clinical Department of Cardiology and Angiology at the University Clinical Centre Maribor, Slovenia, between 2000 and 2022 who had available Lp(a) results at admission. Patients treated with monoclonal antibodies against proprotein convertase subtilisin/kexin type 9 and inclisiran were excluded because of known interference with Lp(a) levels. Most Lp(a) values were collected between 2005 and 2008 when Lp(a) was part of the routine laboratory protocol at admission. Outside this period, Lp(a) was occasionally measured if requested by the attending physician for additional CV risk assessment. Finally, a total of 2248 patients were included in this study. Patients were treated in accordance with the ESC guidelines [23].

The study protocol was approved by the institution’s ethics committee (UKC-MB- KME-8/23; 23 February 2023). The retrospective data analysis was conducted in accordance with the ethical guidelines of the Declaration of Helsinki.

### 2.2. Methods

In all patients, Lp(a) samples were collected within 24 h of admission for incident AMI and analyzed on the same day. Quantitative serum determination of Lp(a) was performed using a fully automated particle-enhanced immunonephelometric assay (Siemens ProSpec, Siemens Healthineers, Erlangen, Germany). Lp(a) results are expressed in mass units (mg/dL). Fasting triglycerides were measured using the enzymatic method, and total serum cholesterol was determined by the cholesterol esterase enzymatic assay. Low-density lipoprotein cholesterol (LDL-C) and high-density lipoprotein cholesterol (HDL-C) were measured using the homogeneous direct method (Siemens Dimension Vista, Healthineers, Erlangen, Germany).

All medical reports concerning recurrent AMI, as well as the time and cause of death, were retrospectively collected from our hospital database and regional referring hospitals until 31 December 2022. The mean observation time was 9.5 years. For all patients who died during the observational period, the time of death and the ICD-10 codes defining the principal cause of death were provided by the National Institute of Public Health, Slovenia.

A recurrent AMI was defined as either a repeat hospitalization with principal ICD-10 discharge codes I21/I22, or when I21/I22 ICD-10 codes were identified as the principal cause of death in the National Mortality Database (National Institute of Public Health, Slovenia). CV death was defined by I00–I99 ICD-10 codes identified as the principal cause of death in the National Mortality Database (National Institute of Public Health, Slovenia).

### 2.3. Statistical Analysis

Categorical variables were summarized using frequencies and percentages. Normally distributed continuous variables are presented as the means ± standard deviations (SDs), whereas nonnormally distributed variables are presented as the medians and corresponding interquartile ranges (IQRs).

Initially, a multivariable Cox regression analysis was performed to analyze the association of Lp(a) levels as a continuous variable with CV events (recurrent AMI, CV death, all-cause death). The model was adjusted for age, sex, LDL-C, HDL-C, triglycerides, statin use, and diabetes mellitus.

Second, a multivariable Cox regression analysis was performed to analyze the association of Lp(a) as an interval variable with CV events (recurrent AMI, CV death, all-cause death). Patients were stratified into three groups based on their Lp(a) level: ≤50 mg/dL, 51–90 mg/dL, and >90 mg/dL. These cutoff values were derived from previous studies and recommendations (2). The model was adjusted for age, sex, LDL-C, HDL-C, triglycerides, statin use, and diabetes mellitus.

Finally, to examine the effect of age and sex on the relationship between Lp(a) and recurrent AMI/mortality, all patients were further stratified based on sex and age into four subgroups with an age threshold of 65 years (men/women aged ≤ 65 years and >65 years). For each age and sex subgroup, a multivariable-adjusted Cox regression analysis was performed to evaluate the impact of Lp(a) as an interval variable on recurrent AMI, CV death, and all-cause death. The model was adjusted for LDL-C, HDL-C, triglycerides, statin use, and diabetes mellitus.

All statistical analyses were performed using SPSS version 28 (IBM, Armonk, NY, USA). A *p*-value < 0.05 was considered statistically significant.

## 3. Results

A total of 2248 patients, with a mean age of 64.7 ± 12.2 years (31.5% women), were included in this study. At the time of inclusion, 24.8% of patients had diabetes mellitus, 9.1% were insulin-dependent, and 10.8% were treated with oral antidiabetic drugs. A statin was prescribed in 82.0% of patients, with 23.5% receiving high-intensity statin therapy (rosuvastatin 20–40 mg, atorvastatin 40–80 mg). Beta-blockers were used in 79.9%, angiotensin-converting enzyme inhibitors in 75.5%, and mineralocorticoid receptor antagonists in 8.1% of patients. During the mean follow-up of 9.5 years, recurrent AMI occurred in 453 patients (20.2%), CV death in 650 (28.9%), and all-cause death in 1199 (53.3%) patients. The mean time to recurrent AMI was 58.5 ± 56.5 months, to CV death was 74.6 ± 58.8 months, and to all-cause death was 82.5 ± 60.3 months. The median Lp(a) level in the cohort was 17.0 mg/dL, with an IQR of 7.0–47.0 mg/dL. The mean LDL-C level was 3.1 ± 1.1 mmol/L, the mean HDL level was 1.1 ± 0.3 mmol/L, and the mean triglyceride level was 1.9 ± 1.2 mmol/L. The mean creatinine level was 91.9 ± 53.3 mcmol/L. The basic characteristics of the included patients are summarized in Table 1.

In the multivariable Cox regression model using Lp(a) as a continuous variable, no significant associations were found between Lp(a) and recurrent AMI (HR 1.00, *p* = 0.171), CV death (HR 1.00, *p* = 0.332), or all-cause death (1.00, *p* = 0.062) (Supplementary Table S1).

**Figure 1:**
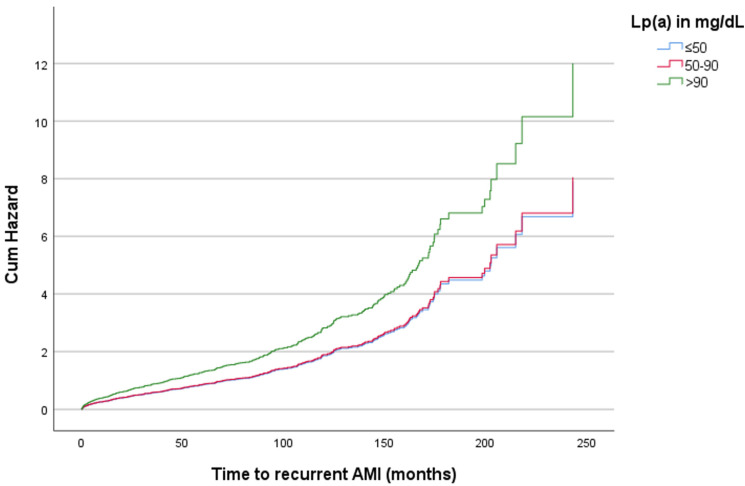
Association of Lp(a) as an interval variable with time to recurrent AMI in the whole cohort. The multivariable-adjusted hazard ratios (HRs) of recurrent AMI were 1.01 (*p* = 0.921, 95% CI 0.768–1.340) for Lp(a) levels between 51 and 90 mg/dL and 1.51 (*p* = 0.013, 95% CI 1.093–2.094) for levels > 90 mg/dL, compared with levels ≤ 50 mg/dL. The Cox regression model was adjusted for age, sex, low-density lipoprotein cholesterol, high-density lipoprotein cholesterol, triglycerides, statin use, and diabetes mellitus. AMI: acute myocardial infarction; Lp(a): lipoprotein(a).

Patients were further stratified into three groups based on their Lp(a) level (≤50, 51–90, >90 mg/dL). The number of included patients and the sex distribution within each Lp(a) group are summarized in Supplementary Table S2. The multivariable-adjusted hazard ratios (HRs) of recurrent AMI were 1.01 (*p* = 0.921, 95% confidence interval [CI]: 0.768–1.340) for levels between 51 and 90 mg/dL and 1.51 (*p* = 0.013, 95% CI: 1.093–2.094) for levels > 90 mg/dL, compared with levels ≤ 50 mg/dL (Table 2, Figure 1). For CV mortality, the HRs were 1.13 (*p* = 0.300, 95% CI: 0.899–1.412) for levels between 51 and 90 mg/dL and 1.14 (*p* = 0.348, 95% CI: 0.869–1.487) for levels > 90 mg/dL, compared with levels ≤ 50 mg/dL. For all-cause mortality, the HRs were 1.09 (*p* = 0.310, 95% CI: 0.923–1.285) for levels between 51 and 90 mg/dL and 1.20 (*p* = 0.090, 95% CI: 0.972–1.477) for levels > 90 mg/dL, compared with levels ≤ 50 mg/dL (Table 2).

**Figure 2:**
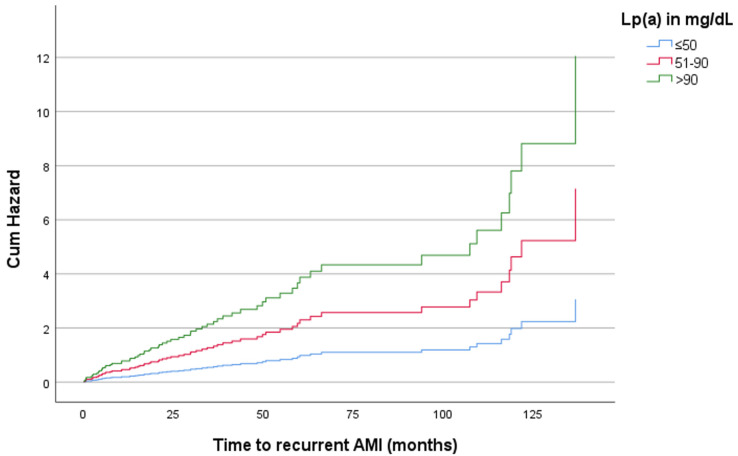
Association of Lp(a) as an interval variable with time to recurrent AMI in women aged > 65 years. After stratification for age and sex, Lp(a) was significantly associated with time to recurrent AMI only in women aged > 65 years, with a multivariable-adjusted HR of 2.34 (*p* = 0.013) for levels between 51 and 90 mg/dL and 3.94 (*p* < 0.001) for levels > 90 mg/dL, compared with levels ≤ 50 mg/dL. The Cox regression model was adjusted for low-density lipoprotein cholesterol, high-density lipoprotein cholesterol, triglycerides, statin use, and diabetes mellitus. AMI: acute myocardial infarction; Lp(a): lipoprotein(a).

After stratification by sex and age, the positive association of Lp(a) with recurrent AMI remained statistically significant only for women aged > 65 years, with a multivariable-adjusted HR of 2.34 (*p* = 0.013, 95% CI: 1.198–4.563) for levels between 51 and 90 mg/dL and 3.94 (*p* < 0.001, 95% CI: 1.760–8.833) for levels > 90 mg/dL, compared with levels ≤ 50 mg/dL (Table 3, Figure 2). No significant associations of Lp(a) with CV or all-cause mortality were found in any age or sex subgroup (Supplementary Tables S3 and S4).

## 4. Discussion

In the presented study, Lp(a) was associated with a significantly higher risk of recurrent AMI only in women aged > 65 years with Lp(a) levels > 50 mg/dL. We found no significant associations between Lp(a) and CV or all-cause mortality.

Most large previous Lp(a) trials included patients in the primary prevention setting, confirming the causal association of Lp(a) with the first CV event [3,4,5,6,24,25,26]. However, data concerning the impact of Lp(a) on recurrent CV events in patients with established ASCVD are more controversial [7,20,21,27]. In 1996, the first study comparing the role of Lp(a) in primary versus secondary prevention confirmed the association of Lp(a) only with the first CV event [28]. In 2014, the first extensive meta-analysis of secondary prevention trials revealed heterogeneous results, showing a significant association between Lp(a) and the second CV event only in patients with LDL-C levels greater than 3.7 mmol/L [20]. Similarly to our findings, a study including STEMI patients found the association of Lp(a) with recurrent events only in patients with an extreme Lp(a) level above the 95th percentile (≥135 mg/dL) [29]. Furthermore, in a very recently published study of 32,537 ASCVD patients, Lp(a) levels > 150 nmol/L versus < 65 nmol/L were associated with an increased risk of recurrent CV events, particularly nonfatal AMI and coronary revascularizations, which was most evident in the first year following the index event [19]. Conversely, in the subanalysis of the dal-Outcome randomized dalcetrapib trial, Lp(a) was not significantly associated with recurrent adverse CV outcomes [21]. In the analysis of the large UK Biobank data encompassing nearly half a million individuals, Lp(a)-associated CV risk was attenuated among those with preexisting ASCVD compared with those without (5% versus 10% higher risk conferred by a 50 nmol/L increase in Lp(a) level) [7,22]. This suggests that in very high-risk ASCVD patients, the relative risk contribution of Lp(a) may be outweighed by other more decisive factors. This is an important consideration in light of emerging Lp(a)-lowering drugs, underscoring the need for further research to identify subgroups that might benefit the most.

The differential impact of age and sex on Lp(a)-associated morbidity and mortality in the general population was recently evaluated using the data from the large Copenhagen general population cohort [12]. When comparing individuals with Lp(a) levels > 40 mg/dL versus < 10 mg/dL, the increase in the risk of incident AMI was similar in both sexes [12]. The same study reported a similar increase in Lp(a)-associated CV risk in those below and above 50 years of age [12]. In a large pooled multiethnic sample from five landmark primary prevention cohorts, the Lp(a)-associated risk of long-term CV events was similar across age, sex, and race/ethnicity [30]. On the other hand, the role of sex and age in patients with prevalent ASCVD is much less clear, and the data are limited. In our study, we found a strong association between Lp(a) levels and recurrent AMI only in women aged > 65 years, with risk increasing beyond an Lp(a) threshold of 50 mg/dL. However, a trend toward increased CV risk associated with Lp(a) levels > 90 mg/dL compared with levels ≤ 50 mg/dL was observed also in women aged ≤ 65 years, although it did not reach statistical significance (HR = 2.64, *p* = 0.150). Interestingly, we identified a significantly lower recurrence of AMI in men with Lp(a) levels between 51 and 90 mg/dL compared with those with levels ≤ 50 mg/dL (HR = 0.45, *p* = 0.046). This finding has not been reported in previous studies, and no clear biological explanation currently exists to account for it [2,3,4,7]. Similarly to our findings, Bigazzi et al. highlighted the differential impact of sex in patients with prevalent coronary artery disease, noting a greater risk of future coronary revascularizations in women with elevated Lp(a) than in men [31]. In contrast, a recent study of 12,064 ASCVD patients following a percutaneous coronary intervention found no sex-related differences in the association between Lp(a) and future CV events [32].

Women have generally 5–10% higher Lp(a) levels compared with men [2,18]. Lp(a) levels tend to increase with advancing age in both sexes, primarily due to an age-related decline in glomerular filtration rate [12]. Additionally, most studies have observed a further modest increase in Lp(a) levels in women after menopause, likely influenced by hormonal changes [2,12,33,34]. Menopause has also been associated with significantly elevated systemic oxidative stress [35,36]. Estradiol is a potent antioxidant, and its decline after menopause contributes to an increased rate of lipid peroxidation and the accumulation of oxidized phospholipids in atherogenic lipoprotein particles, thereby enhancing their endothelial permeability [37,38,39]. This aligns with our observations, which suggest that the atherogenic potential of Lp(a) may be higher in postmenopausal compared with premenopausal women, warranting further research.

The association of Lp(a) with CV and all-cause mortality is another unresolved issue. While some studies aligned with our findings and reported no association, others identified Lp(a) as a risk factor for increased CV or all-cause mortality in the general population [40,41,42,43]. In 2009, the Emerging Risk Factors Collaboration meta-analysis demonstrated that one standard deviation higher Lp(a) was associated with significantly higher CV mortality (HR 1.14, 95% CI: 1.07–1.22), whereas all-cause mortality was not analyzed [4]. In a large cohort of the Danish general population, a 50 mg/dL (105 nmol/L) increase in Lp(a) level was associated with an HR of 1.16 for CV mortality (95% CI: 1.09–1.23) and 1.05 (95% CI: 1.01–1.09) for all-cause mortality, which was further confirmed by genetic associations on the basis of the number of *LPA* kringle 4 type-2 repeats and the *LPA* rs10455872 genotype [44]. In patients with prevalent coronary heart disease, a large genetic association study revealed no associations between Lp(a) levels/corresponding genetic variants and long-term mortality, which is consistent with our findings [45]. Intriguingly, a Japanese general population study of 10,413 individuals showed paradoxically higher all-cause mortality in patients with low (<8 mg/dL) versus intermediate–high Lp(a) (≥8 mg/dL) (HR 1.43, 95% CI: 1.21–1.68) [46]. This could at least partly be explained by the higher incidence of cancer-related deaths observed in the group with low Lp(a) levels in their study, while the potential antineoplastic properties of Lp(a) are an area of research [46]. Furthermore, increased Lp(a) might protect against major bleeding due to its structural homology with plasminogen and potential antifibrinolytic properties. This hypothesis was recently tested by Langsted A et al. in Danish general population study participants, demonstrating that one standard deviation increase in Lp(a) (31 mg/dL) was associated with a decreased hazard for major bleeding in the brain and airways (HR 0.95, 95%: CI 0.91–1.00), which was further confirmed by a genetic association study [47].

Our results should be interpreted in light of certain limitations. First, the analysis was retrospective and relied on the ICD-10 diagnoses reported at hospital discharge by the discharging physician. The retrospective approach inherently limits our ability to control for unrecorded variables, including inflammatory markers like the high-sensitivity C-reactive protein. The absence of genetic data restricts our ability to establish causality between Lp(a) levels and the outcomes observed in this study. Additionally, Lp(a) measurements were conducted only on a limited number of patients admitted for AMI during the study period, which may introduce potential selection bias.

The major strengths of our study are the inclusion of a homogeneous Caucasian (Slovenian) population and the consistency in Lp(a) measurements on the day of blood sampling, thereby avoiding potential measurement errors due to delayed sample analysis [48]. Moreover, all the measurements were conducted using the same laboratory method (nephelometry) and were performed with Siemens laboratory equipment.

## 5. Conclusions

Lp(a) is a well-established risk factor for incident CV events, particularly AMI. However, its role in patients with established ASCVD is less clear. In the presented study, Lp(a) was associated with a significantly higher risk of recurrent AMI only in women aged > 65 years with Lp(a) levels > 50 mg/dL, and no associations were observed between Lp(a) levels and CV or all-cause mortality.

## Figures and Tables

**Table 1:** Patient characteristics (N = 2248).

Clinical Variable	Value
Age (years, mean ± SD)	64.7 ± 12.2
Women (%)	31.5
Diabetes mellitus (%)	24.8
Arterial hypertension (%)	60.0
STEMI/NSTEMI (%)	36.7/63.3
**Laboratory data**	**Mean ± SD**
Hemoglobin (g/L)	135.0 ± 17.5
Thrombocytes (10^9^/L)	226.1 ± 74.5
HDL-C (mmol/L)	1.1 ± 0.3
Triglycerides (mmol/L)	1.9 ± 1.2
LDL-C (mmol/L)	3.1 ± 1.1
Creatinine (mcmol/L)	91.9 ± 53.3
Uric acid (mcmol/L)	327.1 ± 107.3
**Medication**	**Percent (%)**
Statins (no statin/moderate/high intensity)	18.0/58.5/23.5
Ezetimibe	1.2
Fenofibrate	2.2
Insulin	9.1
Oral antidiabetic drugs	10.8
Beta-blockers	79.9
Angiotensin-converting enzyme inhibitors	75.5
Angiotensin II receptor blockers	12.4
Mineralocorticoid receptor antagonists	8.1
Calcium channel antagonists	12.9
Alpha-adrenergic receptor antagonists	2.8

High-intensity statins included rosuvastatin 20–40 mg and atorvastatin 40–80 mg. Lower doses of rosuvastatin and atorvastatin, as well as other statins used by study participants (such as simvastatin, pravastatin, and fluvastatin), were classified as low to moderate intensity. STEMI: myocardial infarction with ST-segment elevation; NSTEMI: myocardial infarction without ST-segment elevation; HDL: high-density lipoprotein cholesterol; LDL-C: low-density lipoprotein cholesterol.

**Table 2:** Association of Lp(a) as an interval variable with recurrent AMI and mortality.

Lp(a) in mg/dL	Hazard Ratio	*p*-Value	95% CI
Recurrent AMI			
≤50	Reference		
51–90	1.01	0.921	0.768–1.340
>90	1.51	0.013	1.093–2.094
CV mortality			
≤50	Reference		
51–90	1.13	0.300	0.899–1.412
>90	1.14	0.348	0.869–1.487
All-cause mortality			
≤50	Reference		
51–90	1.09	0.310	0.923–1.285
>90	1.20	0.090	0.972–1.477

All patients were stratified into three groups on the basis of their Lp(a) level: ≤50 mg/dL, 51–90 mg/dL, and >90 mg/dL. Lp(a) > 90 mg/dL was significantly associated with recurrent AMI. Lp(a) was not associated with CV or all-cause mortality. AMI: acute myocardial infarction; CI: confidence interval; CV: cardiovascular; Lp(a): lipoprotein(a).

**Table 3:** Association of Lp(a) as an interval variable with recurrent AMI stratified by age and sex.

Sex	Age (Years)	Lp(a) (mg/dL)	Hazard Ratio	*p*-Value	95% CI
Men	≤65	≤50	Reference		
		51–90	1.26	0.354	0.773–2.054
		>90	1.06	0.839	0.597–1.887
	>65	≤50	Reference		
		51–90	0.45	0.046	0.201–0.986
		>90	1.18	0.681	0.529–2.646
Women	≤65	≤50	Reference		
		51–90	0.29	0.114	0.064–1.343
		>90	2.64	0.150	0.704–9.868
	>65	≤50	Reference		
		51–90	2.34	0.013	1.198–4.563
		>90	3.94	<0.001	1.760–8.833

Lp(a) was positively associated with recurrent acute myocardial infarction in women aged > 65 years and Lp(a) > 50 mg/dL. CI: confidence interval; Lp(a): Lipoprotein(a).

## Data Availability

The data that support the findings of this study are available from the corresponding author upon a reasonable request. The data are not publicly available due to ethical concerns.

## Supplementary Materials

The following supporting information can be downloaded at: https://www.mdpi.com/article/10.3390/diagnostics14232757/s1, Table S1. Association of Lp(a) as a continuous variable (in mg/dL) and other covariates with recurrent AMI and mortality; Table S2. Number and sex distribution of patients stratified by Lp(a) level in three groups; Table S3. Association of Lp(a) as an interval variable with CV mortality stratified by age and sex; Table S4. Association of Lp(a) as an interval variable with all-cause mortality stratified by age and sex.

## List of Abbreviations

AMI	acute myocardial infarction
ASCVD	atherosclerotic cardiovascular disease
CV	cardiovascular
HDL-C	high-density lipoprotein cholesterol
ICD-10	Tenth Revision of the International Classification of Diseases diagnosis codes
IQR	interquartile range
LDL-C	low-density lipoprotein cholesterol
Lp(a)	lipoprotein(a)
NSTEMI	myocardial infarction without ST-segment elevation
SD	standard deviation
STEMI	myocardial infarction with ST-segment elevation
