# Supplementary material for: Lipoprotein(a) as a Risk Factor for Recurrent Acute Myocardial Infarction and Mortality: Insights from Routine Clinical Practice
Source: Diagnostics (Basel). 2024 Dec 7;14(23):2757. doi: 10.3390/diagnostics14232757 (PMC11640375; doi:10.3390/diagnostics14232757)
Supplement: Supplementary file 1 [file diagnostics-14-02757-s001.zip › diagnostics-3283672-supplementary.pdf]

SUPPLEMENTARY TABLES

Table S1. Association of Lp(a) as a continuous variable (in mg/dL) and other covariates with recurrent acute myocardial infarction and mortality

Variable (N=2248)	Hazard ratio	*p*-Value
**Recurrent AMI**		
Age	1.03	<0.001
Sex	1.21	0.078
Lp(a)	1.00	0.171
HDL-C	0.99	0.954
Triglycerides	0.94	0.168
LDL-C	0.93	0.162
Statins	0.82	0.340
Diabetes	1.10	0.365
**CV mortality**		
Age	1.40	<0.001
Sex	1.11	0.233
Lp(a)	1.00	0.332
HDL-C	0.82	0.142
Triglycerides	0.94	0.121
LDL-C	0.89	0.010
Statins	0.95	0.563
Diabetes	1.35	<0.001
**All-cause mortality**		
Age	1.73	<0.001
Sex	1.10	0.133
Lp(a)	1.00	0.062
HDL-C	0.84	0.071
Triglycerides	0.95	0.087
LDL-C	0.90	0.001
Statins	0.89	0.088
Diabetes	1.29	<0.001

Lp(a) as a continuous variable was not significantly associated with recurrent AMI, CV death, or all-cause death. AMI: acute myocardial infarction, CV: cardiovascular, HDL: high-density lipoprotein cholesterol, LDL-C: low-density lipoprotein cholesterol, Lp(a): lipoprotein(a).

Table S2. Number and sex distribution of patients stratified by Lp(a) level in three groups

Lp(a) group (mg/dL)	Men	Women	Total
≤50	1,198	506	1,704
51–90	222	116	338
>90	120	86	206

All patients were stratified into three groups based on their Lp(a) level: ≤ 50 mg/dL, 51–90 mg/dL, and > 90 mg/dL. Lp(a): lipoprotein(a).

Table S3: **Association of Lp(a) as an interval variable with CV mortality stratified by age and sex**

Sex	Age (years)	Lp(a) in mg/dL	Hazard ratio	*p*-Value
Men	≤65	≤50	Reference	
		51–90	1.22	0.246
		>90	1.43	0.084
	>65	≤50	Reference	
		51–90	1.05	0.728
		>90	1.26	0.242
Women	≤65	≤50	Reference	
		51–90	0.53	0.106
		>90	0.62	0.326
	>65	≤50	Reference	
		51–90	0.96	0.779
		>90	1.16	0.397

Patients were stratified into three groups based on their Lp(a) level: ≤ 50 mg/dL, 51–90 mg/dL, and > 90 mg/dL. Lp(a) was not significantly associated with CV death in either men or women. CV: cardiovascular, Lp(a): lipoprotein(a).

Table S4: **Association of Lp(a) as an interval variable with all-cause mortality stratified by age and sex**

Sex	Age (years)	Lp(a) in mg/dL	Hazard ratio	*p*-Value
Men	≤65	≤50	Reference	
		51–90	1.22	0.246
		>90	1.43	0.084
	>65	≤50	Reference	
		51–90	1.05	0.728
		>90	1.26	0.242
Women	≤65	≤50	Reference	
		51–90	0.53	0.106
		>90	0.622	0.326
	>65	≤50	Reference	
		51–90	1.011	0.951
		>90	1.124	0.583

Patients were stratified into three groups based on their Lp(a) level: ≤ 50 mg/dL, 51–90 mg/dL, and > 90 mg/dL. Lp(a) was not significantly associated with all-cause death in either men or women. Lp(a): lipoprotein(a).
